# Venom trade-off shapes interspecific interactions, physiology, and reproduction

**DOI:** 10.1126/sciadv.adk3870

**Published:** 2024-03-13

**Authors:** Joachim M. Surm, Sydney Birch, Jason Macrander, Adrian Jaimes-Becerra, Arie Fridrich, Reuven Aharoni, Rotem Rozenblat, Julia Sharabany, Lior Appelbaum, Adam M. Reitzel, Yehu Moran

**Affiliations:** ^1^ Department of Ecology, Evolution and Behavior, Alexander Silberman Institute of Life Sciences, Faculty of Science, The Hebrew University of Jerusalem, Jerusalem, Israel.; ^2^ Department of Biological Sciences, University of North Carolina at Charlotte, Charlotte, NC, USA.; ^3^ Biology Department, Florida Southern College, Lakeland, FL, USA.; ^4^ Faculty of Life Sciences, Bar-Ilan University, Ramat Gan, Israel.; ^5^ The Multidisciplinary Brain Research Center, Bar-Ilan University, Ramat Gan, Israel.

## Abstract

The ability of an animal to effectively capture prey and defend against predators is pivotal for survival. Venom is often a mixture of many components including toxin proteins that shape predator-prey interactions. Here, we used the sea anemone *Nematostella vectensis* to test the impact of toxin genotypes on predator-prey interactions. We developed a genetic manipulation technique to demonstrate that both transgenically deficient and a native *Nematostella* strain lacking a major neurotoxin (Nv1) have a reduced ability to defend themselves against grass shrimp, a native predator. In addition, secreted Nv1 can act indirectly in defense by attracting mummichog fish, which prey on grass shrimp. Here, we provide evidence at the molecular level of an animal-specific tritrophic interaction between a prey, its antagonist, and a predator. Last, this study reveals an evolutionary trade-off, as the reduction of Nv1 levels allows for faster growth and increased reproductive rates.

## Teaser

Venom shapes interspecific interactions between sea anemones and predators and mediates a trade-off with growth and reproduction.

## INTRODUCTION

Capturing prey and defending against predators are vital tasks required for animal survival. During evolution, animals have effectively optimized their predator and prey interactions while also balancing other traits essential for maintaining fitness such as gamete production and growth. As resources are limited, there is often a trade-off between the energy an organism uses for each trait, which leads to a diversity of phenotypes observed at the individual, population, and species levels (*1*). Uncovering the evolutionary trade-off between metabolically costly traits is, therefore, essential to understand its impact on the phenotype and fitness of the organism. Understanding the genetic basis of a trait is essential for unraveling the molecular processes responsible for driving the phenotypic changes being selected; however, connecting this genotype-phenotype link is challenging as most traits are shaped by many genes that often have multiple functions.

Having venom is a trait involved in predator-prey interactions and is known to evolve under strong positive selection pressures (*2*). Venomous animals deploy a complex mixture of molecules to cause a physiological imbalance in another animal (*2*). A major component of venom are toxic proteins, henceforth referred to as toxins, which are directly encoded by the venomous animal’s genome. These toxin-encoding genes have been shown to evolve under strong selective pressure at both the sequence and gene expression levels (*3*–*5*), due to their important ecological implications as well as their likely high metabolic cost of production (*6*, *7*). Furthermore, toxins are directly involved in predator-prey interactions and have been shown to have specialized functional roles. Notable examples of this specialized role of venom have been shown in multiple animals including scorpions, cone snails, and the assassin bug (*Pristhesancus plagipennis*), which can also dynamically shift their venom profile in response to predation or defense-evoked stimulation (*8*–*10*). Because of these factors, venom is an excellent system for understanding functional adaptations at the molecular level by connecting the toxin genotype to the ecological phenotype, such as predation or defense.

**Fig. 1.**
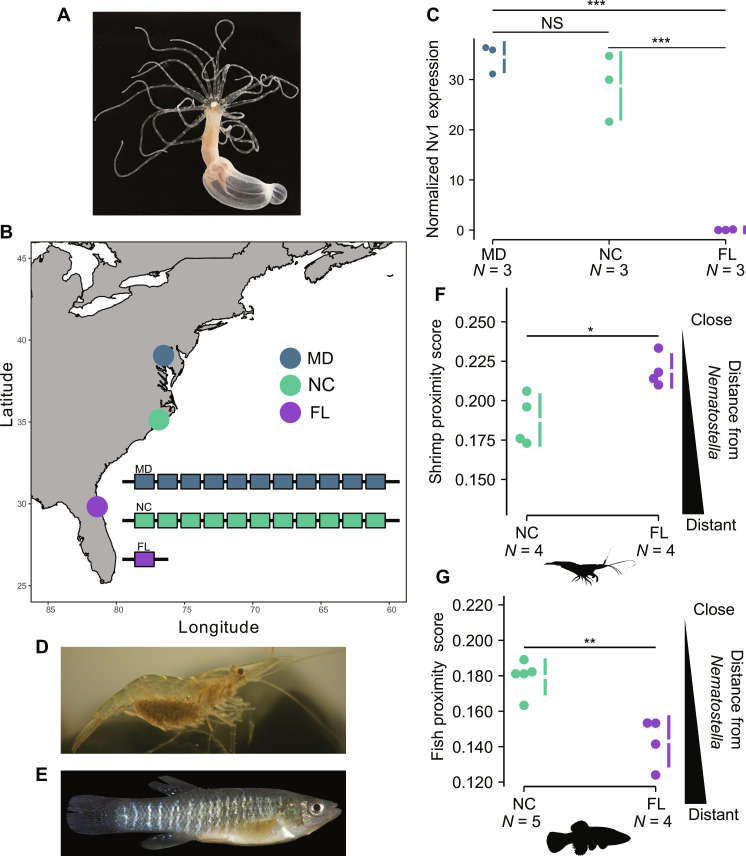
Population variation of *Nv1* and its impact on defense against predators. (**A**) *Nematostella vectensis*. (**B**) Map showing the location of the different populations of *Nematostella* across North America. Inset, maximum haploid copy number of *Nv1* reported in different *Nematostella* populations. (**C**) nCounter relative RNA expression levels of *Nv1* from different populations. (**D**) Grass shrimp (*Palaemonetes pugio*). Picture taken from Wikimedia by Brian Gratwicke (2006). (**E**) Mummichog (*Fundulus heteroclitus*). Picture taken from Wikimedia from the Smithsonian Environmental Research Center. (**F**) Average weighted score of grass shrimp proximity to *Nematostella* from NC and FL. (**G**) Average weighted score of mummichog proximity to *Nematostella* from NC and FL. Confidence intervals (95%) are indicated by the ends of the vertical error bars. *P < 0.05, **P < 0.01, ***P < 0.001; NS, not significant. Species silhouettes were sourced from PhyloPic. Grass shrimp (*P. pugio*) and Mummichog (*F. heteroclitus*) picture taken from Wikimedia. *Nematostella* photo credit: Yael Admoni.

The sea anemone *Nematostella vectensis (*Fig. 1A) is arguably the most developed venomous model system, being amendable to genetic manipulation (*11*) and having much of the venom previously characterized (*12*). In *Nematostella*, Nv1 is a neurotoxin that modulates voltage-gated sodium channels by inhibiting their inactivation, making it highly lethal to various arthropods (*13*, *14*). Nv1 is the dominant toxin in adult venom, being produced in massive amounts in ectodermal gland cells, and is among the most abundant proteins in the entire whole-animal proteome (*4*, *13*, *15*). The abundance of the protein is due to the *Nv1* locus having more than 10 copies in a tandem array that all produce the same mature protein (*4*, *16*). Our previous work revealed that *Nv1 *copies are abundant in the genome, with all populations surveyed having more than 10 diploid copies (*4*). The exception, however, is a population originating from Florida (FL), which has probably undergone a contraction event resulting in only a single diploid copy (Fig. 1B). This contraction event resulted in a significant reduction of *Nv1* at the transcriptional level compared to other populations, including the North Carolina (NC) population is genetically close to FL (*4*), as well as the farther Maryland (MD) population [Fig. 1C and table S1; analysis of variance (ANOVA) *P* = 0.0001], which is the source of the common *N. vectensis* laboratory strain (*11*, *17*). At the protein level, Nv1 was undetectable in FL compared to being among the top five most abundant protein from NC *Nematostella* whole-animal proteome (*4*). We find that the loss of *Nv1* in FL is not an anecdotal phenomenon, with additional sampling of *Nematostella* 12 km from the original site, revealing that all individuals (*n* = 10) have 0 to 2 *Nv1* diploid copies (table S1). While this previous work characterized the molecular signature that underlies *Nv1* variation across populations (*4*), how this variation affects key life history characteristics such as predator-prey interactions and reproduction remains to be tested. Such insights would allow us to connect how changes in a genotype can affect the fitness of an animal and any potential trade-offs that exist.

To unravel how a toxin genotype can affect the venom phenotype and affect an individual’s fitness, we investigated the impact of losing a toxin at the organismal level using a combination of behavioral and organismal assays. We developed a genetic manipulation technique using transgenesis to constitutively knock down all *Nv1* copies and challenged *Nematostella* of this transgenic strain as well as those of the native population from FL to capture prey and defend themselves against predators. We find that the FL population and the transgenic knockdown line, which both have depleted Nv1 levels, phenocopy and have a significantly reduced capacity to defend themselves against native predatory shrimp compared to *Nematostella* with high levels (henceforth referred to as wild-type levels) of Nv1. In contrast, when we test these animals against native fish known to predate on *Nematostella* larvae, we find that *Nematostella* adult polyps that produce wild-type amounts of Nv1 attract the fish. Further experiments reveal that indeed Nv1 is being continuously secreted by *Nematostella* into the water and is detected by fish, suggesting that it is acting as an attractant. Last, when testing the physiological impact of synthesizing the massive amounts of Nv1, we find that *Nematostella* with depleted Nv1 levels grow faster and are more reproductive, highlighting that Nv1 synthesis is implicated in a significant evolutionary trade-off with key fitness-related characters.

## RESULTS

### Population variation of *Nv1* and its impact on interspecific interactions

We have set out to experimentally test the impact of losing Nv1 in predator-prey interactions. We first tested the ability of *Nematostella* to defend itself against ecologically relevant predators (*15*, *18*, *19*), specifically grass shrimp (Fig. 1D; *Palaemonetes pugio*) and mummichogs (Fig. 1E; *Fundulus heteroclitus*), all of which have a distribution that spans across the Atlantic coast of North America (*18*, *20*, *21*). We hypothesized that FL *Nematostella* that have reduced levels of Nv1 would have more difficulty defending themselves against these known predators. To test this, we recorded the interactions between *Nematostella* and each predator independently in a small vessel and scored the location of the predator on the basis of its distance. *Nematostella* individuals that received a low score were considered to have been able to defend themselves as the predator avoided the sea anemone and kept farther away (distant), whereas a high proximity score reflected *Nematostella’s* reduced ability to defend themselves as the predator was more often close to the sea anemone. This method was adapted from previous work (*15*). Our results reveal that *Nematostella* originating from FL have a reduced capacity to defend themselves against grass shrimps, receiving a higher score compared to *Nematostella* from NC (Fig. 1F and table S2; *P* = 0.016). While this is a strong indication that the differences between these populations are the result of a loss of Nv1 in FL, other local adaptations could have occurred that underlie these differences. Therefore, we aimed to genetically manipulate the laboratory strain of *Nematostella*, coming from MD, to target Nv1 specifically, and test whether it is essential for *Nematostella* to defend itself against predators.

### Unraveling the evolutionary trade-off of Nv1 using genetic approaches

**Fig. 2.**
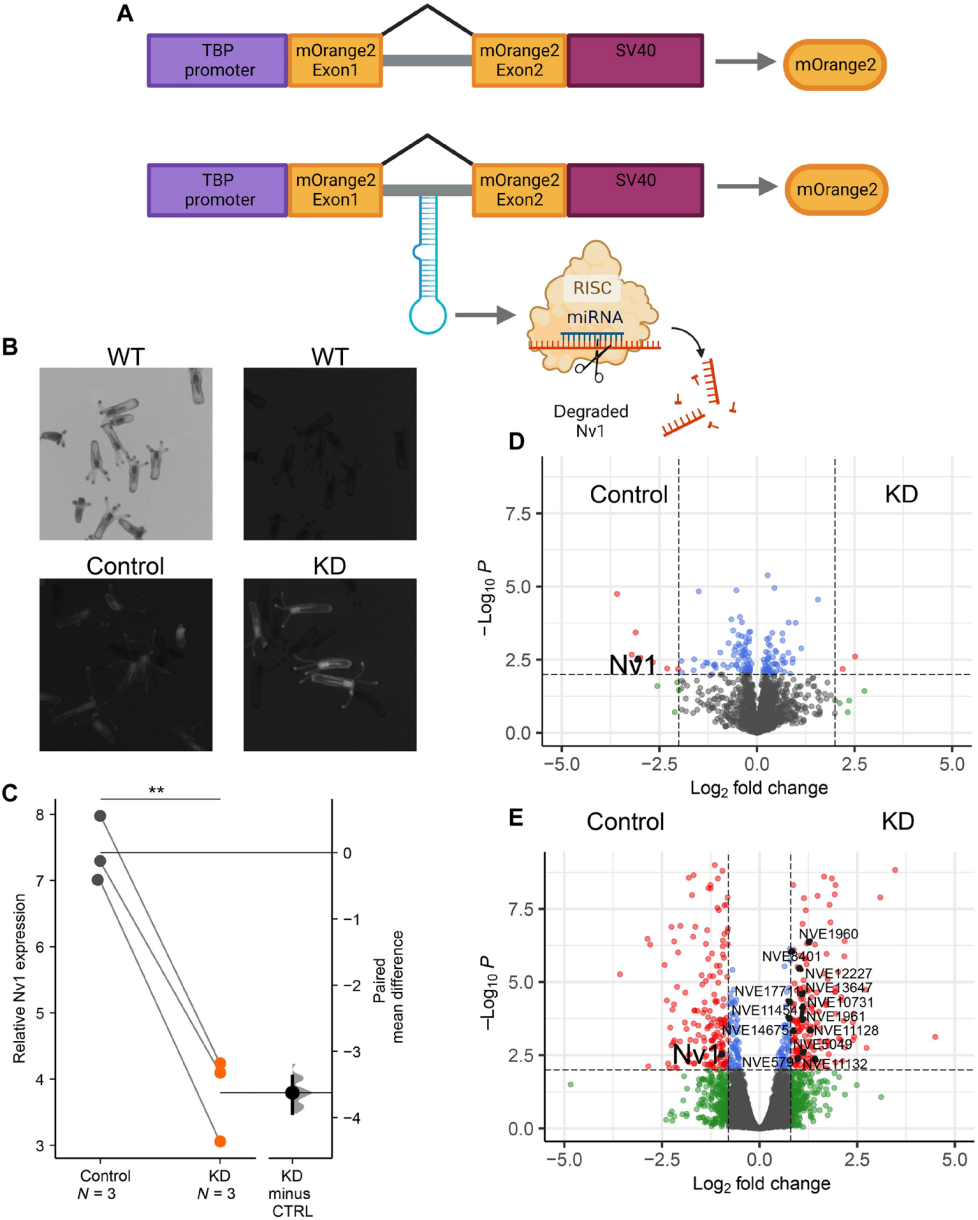
Characterization of the transgenic approach to constitutively knockdown *Nv1*. (**A**) Schematic description of the vector used for transgenesis of both the control and knockdown line. A 2.9-kb TBP promoter was inserted upstream of the mOrange2 gene. Intron taken from NVE19315 (scaffold_507:85,148-86,273). The simian virus 40 (SV40) polyadenylation signal. KD construct also includes a short hairpin designed to target *Nv1*. Created with BioRender.com. (**B**) F_2_ animals showing successfully integration of both constructs compared nonfluorescent wild-type (WT) animals. (**C**) RT–quantitative PCR measuring the expression of *Nv1*. Plotted values are mean relative expression of *Nv1* ΔCT comparing control to KD and represented as Gardner-Altman estimation plot. The mean difference is plotted on a floating axis on the right as a bootstrap sampling distribution. The mean difference is depicted as a dot; the 95% confidence interval is indicated by the ends of the vertical error bar. (**D**) Volcano plot representing proteins of significantly different abundance, measured as label-free quantification (LFQ) intensity, between control and KD. Gray dots represent proteins that are not significant, blue dots are proteins with significant *P* value <0.01, green dots are proteins with Log_2_ fold change of >2, and red dots are proteins with significant *P* value and log_2_ fold change. (**E**) Differentially expressed genes from 2-month-old animals. Differentially expressed genes (red) were defined by false discovery rate (FDR) < 0.01 and fold change ≥1.8. Genes highlighted in black include *Nv1* and those genes found to be enriched in terms related to cell cycle and mitosis. **P < 0.01.

Because Nv1 is encoded by numerous gene copies the CRISPR-Cas9 system cannot be used to delete it from the genome. Thus, to target the unique locus of *Nv1*, we successfully developed an approach to simultaneously and constitutively knocking down all copies of *Nv1*, referred to as KD. This approach relies on the cnidarian microRNA (miRNA) pathway, a specialized RNA interference pathway, that has been shown previously to effectively silence genes in *Nematostella* (*22*, *23*). We generated two lines using meganuclease to randomly integrate a construct that included a short hairpin that targets *Nv1* (KD) and a control transgenic line (control) that included the same features as the KD construct, except for the absence of the short hairpin (Fig. 2A). Both constructs successfully integrated into the *Nematostella* genome and are able to pass on the transgene across generations (Fig. 2B). Using an end-point stem-loop polymerase chain reaction (PCR), we confirmed that the miRNA undergoes proper processing by the small RNA machinery (fig. S1). This approach yielded KD *Nematostella* that have a significant reduction of ~90% of Nv1 at both the RNA (Fig. 2C and table S4; *P* = 0.004) and protein levels (Fig. 2D and table S5; *P* = 0.0027). Overall, these findings support the successful generation of the first genetically modified animal with a reduced venom arsenal.

### The role of Nv1 in defense

**Fig. 3.**
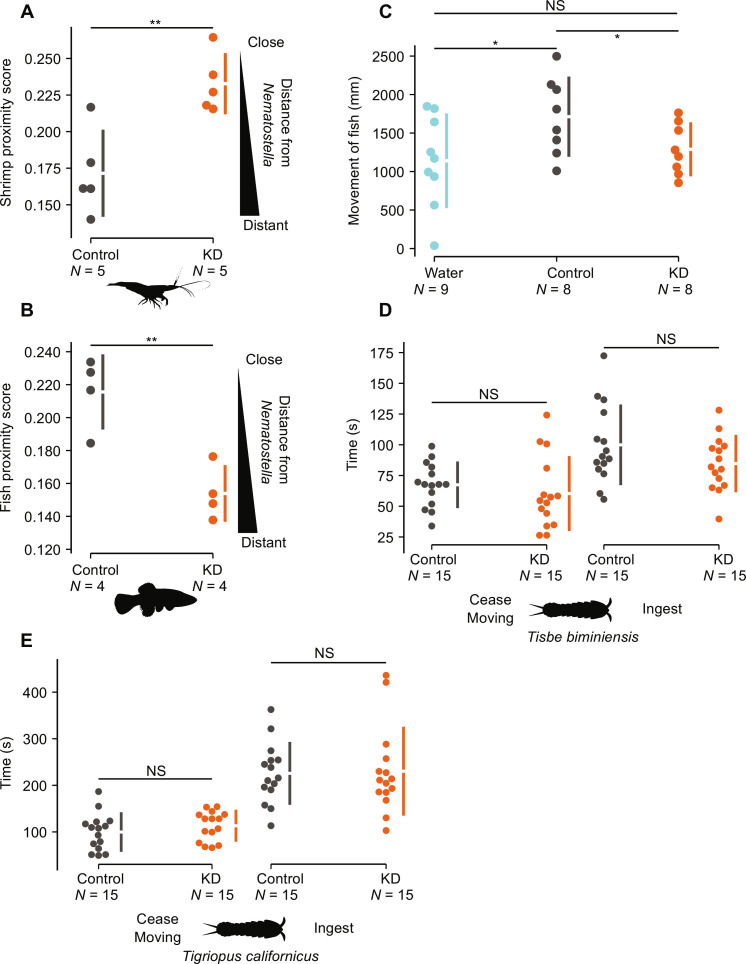
Impact of the depletion of Nv1 on defense and predation in *Nematostella*. (**A**) Weighted score of grass shrimp proximity to *Nematostella* control and KD lines. Confidence intervals (95%) are indicated by the ends of the vertical error bars. (**B**) Weighted score of mummichog proximity to *Nematostella* control and KD lines. Confidence intervals (95%) are indicated by the ends of the vertical error bars. (**C**) Tracking the movement of mummichogs in treated water coming from KD, control or 15‰ ASW and performing pairwise comparison. (**D**) Time taken for *Nematostella* to subdue movement and ingest *Tisbe biminiensis*. (**E**) Time taken for *Nematostella* to subdue movement and ingest *Tigriopus californicus*. Confidence intervals (95%) are indicated by the ends of the vertical error bars. *P < 0.05, **P < 0.01. Species silhouettes were sourced from PhyloPic.

After the successful generation of *Nematostella* transgenic lines with reduced levels of Nv1, we aimed to experimentally test the role and significance of Nv1, specifically in interspecific interactions. We repeated our interspecific interaction assay with known predators, as described above, comparing our control with the KD line. Notably, when we test the interaction of grass shrimp with transgenic *Nematostella*, we find that the KD animals phenocopy the FL animals, with KD animals receiving a significantly higher score than the control line animals (Fig. 3A and table S8; Tukey post hoc test *P* = 0.007). This can be seen in the video footage, which shows the shrimp exhibiting a strong aversion to being touched by a control line animal (movie S1) as well as exhibiting a clear avoidance swimming pattern around the control line animals (movie S2). In contrast, shrimps exhibited a significantly reduced avoidance pattern, often touching and walking over the KD animals (movie S3). In parallel, we tested two additional lines: the wild-type laboratory strain (WT) and the *TBP::mCherry* line (*24*). This was performed because the meganuclease approach for generating transgenics causes random genomic integration and can result in differences in the number of integrations, which may affect the metabolic cost of synthesizing the fluorophore. The two additional lines provide a control for the lack of metabolic burden of an added transgene (WT) and a very bright line likely producing a very large amount of transgene (*TBP::mCherry*). No differences were observed between the WT, *TBP::mCherry*, and control lines, and all were significantly different from the KD line. Moreover, this result agrees with the interpretation that the difference in shrimp behavior is due to a lack of Nv1 and not the transgene itself. This supports the findings that KD and FL animals have a reduced capacity to defend themselves against grass shrimp and provides strong evidence that Nv1 is essential in deterring this invertebrate predator. Furthermore, this confirms that a single venom component is essential for defense.

In contrast, the interaction of the different *Nematostella* lines with mummichog fish, a known native predator of *Nematostella* larvae (*15*, *19*), we find the inverse relationship (Figs. 1G and 3B), compared to the grass shrimp. Specifically, we find that KD *Nematostella* (Fig. 3B and table S9; *P* = 0.004) phenocopy FL population (Fig. 1G and table S3; *P* = 0.006), as they both receive significantly lower scores, with mummichogs swimming at a greater distance to these *Nematostella* compared to the control line and NC *Nematostella*, respectively. This led us to hypothesize that Nv1 is acting as a possible attractant, as the mummichogs are swimming in greater proximity to *Nematostella* with wild-type levels of Nv1 (control line and NC sea anemones). An alternative nonexclusive explanation is that the sharp reduction in Nv1 production in KD and FL animals leads to compensation by overexpression of other venom components that deter mummichogs. However, both RNA sequencing (RNA-seq) experiments and proteomics failed to identify the up-regulation of other known toxins (Fig. 2, D and E, and tables S5 and S6).

We next suspected that for Nv1 to act as a possible attractant for mummichogs, it would need to be continually released into the water. This is supported by previous studies which showed that Nv1 is found in massive amounts in ectodermal gland cells (*15*, *25*). Therefore, we first tested whether the Nv1 protein could be captured at measurable levels from water incubated with *Nematostella*. To do this, we incubated wild-type *Nematostella* overnight in 15‰ artificial seawater (ASW) and filtered it to remove any debris and nematocytes, referred to as treated water. The treated water was then concentrated using ultracentrifugal filters and characterized by quantitative proteomics, revealing that indeed Nv1 is secreted into the water and is among the top 10 most abundant proteins (table S10). The high levels of Nv1 released into the water would therefore allow other animals to encounter it without direct contact with the sea anemone.

We next tested whether treated water alone, coming from the control or KD lines, could affect mummichog behavior. To do this, we incubated two *Nematostella* per well overnight in six-well plates and again filtered to remove any debris and nematocytes. Mummichogs were placed inside the wells with treated water, and their movement was recorded (Fig. 3C and table S11). Notably, we find that mummichogs placed in water incubated with control line animals moved significantly more than the KD line (*P* = 0.03) or filtered 15‰ ASW (*P* = 0.025). This confirms that Nv1 is released into the water continuously and significantly affects mummichog behavior. Together with our previous results showing that mummichogs are attracted to animals producing wild-type levels of Nv1, it is likely that Nv1 attracts mummichogs to *Nematostella*.

While this result was unexpected, previous research by Kneib (*26*) showed that when mummichogs, grass shrimp, and *Nematostella* were all placed in the same tank for 10 weeks, *Nematostella* numbers increased. In contrast, Kneib also found that *Nematostella* numbers decreased when only grass shrimp and *Nematostella* were placed together for 10 weeks. In addition, there is evidence that mummichogs prey on grass shrimps (*26*). These results suggest that attracting mummichogs likely aids adult *Nematostella* in defending against grass shrimps. To understand this result in greater detail, we performed additional behavioral assays in zebrafish, as this is arguably the most well-established fish model system for behavioral studies (*27*, *28*). Although mummichogs and zebrafish are only distantly related, our aim was to test whether some functional adaptation has occurred in mummichogs, allowing it to detect low levels of Nv1 in the water or to dissect the behavior Nv1 has on fish at a greater granularity. This is reasonable as previous work has shown that Nv1 is a highly stable and functional protein in fresh water, even being lethal to zebrafish at high concentrations (*14*). In this assay, we tested the effect of treated water coming from the control line and KD animals, at twice the concentration used for the mummichogs, on 14-day-old zebrafish (*Danio rerio*) and found no significant difference in their locomotive activity under alternating dark and light conditions (fig. S3 and table S12). This suggests that some coevolution may have occurred between *Nematostella* and the mummichogs, which allows the latter to sense Nv1 at low concentrations and cues an attraction to the sea anemone.

### The role of Nv1 in predation

We further tested the role of Nv1 in predation, as intraspecific venom variation in snakes has mostly been linked to differences in diet (*29*–*31*). First, we explored the diets of native *Nematostella* across different populations using a metagenomic approach. We examined cytochrome c oxidase subunit 1 mitochondrial gene (CO1) sequences from the gut contents of *Nematostella* during March, June, and September and in populations spanning the Atlantic coast of North America (Nova Scotia, Maine, New Hampshire, Massachusetts and NC). The number of paired CO1 sequences varied greatly among the individuals at each location. For example, in March, the Massachusetts population contained a single individual with 2789 CO1 sequences (71% from that site alone), whereas another individual had no CO1 sequences recovered. All samples collected in March contained at least one individual lacking CO1 sequences, with the Nova Scotia population having the most individuals lacking CO1 sequences (3 of 10; table S13). This was even more pronounced in June and September, when the majority of individuals (43 of 60) across all locations returned no CO1 sequences, recovering only a total of 139 and 10 CO1 sequences, respectively (table S13). Sequence abundance is indicative of the amount and time since recent predation events (*32*). Our results highlight that food abundances varies significantly across seasons and among individuals, with periods of starvation being common. In addition, we confirmed that arthropods are a major food source for most populations (four of five; fig. S4 and table S8). *Nematostella* are largely opportunistic feeders with a large variety of potential prey items. This is consistent with previous work that focused on *Nematostella* from Nova Scotia which also identified that arthropods are a major food source for *Nematostella* but are ultimately opportunistic (*18*).

To determine the role and significance of Nv1 in capturing prey, we focused on using copepods, which are among the top five most abundant arthropods found in the diet of *Nematostella* across all populations. Using two different copepod species, *Tigriopus californicus* and *Tisbe biminiensis*, we recorded the amount of time for the copepods to cease moving after contact with *Nematostella* and the amount of time for *Nematostella* to ingest after the first contact. Overall, we find no significant difference between the control and KD lines in either metric of predation for both species of copepod (Fig. 3, D and E, and table S14). We further tested the predation success of these lines in capturing young mummichogs but again found no differences (table S15). Together, these results suggest that Nv1 likely plays a minor role, if any, in prey capture.

### The impact of Nv1 production on physiology and reproduction

Next, we hypothesized that the production of Nv1 has a high metabolic cost and therefore would affect the fitness of the organism, especially when resources are limited. Nv1 is produced in massive amounts at the protein level (*15*), and previous work has shown that it is metabolically expensive to replenish the entire venom system of *Nematostella* after depletion (*7*). *Nv1* was among the most highly expressed genes during venom replenishment, further supporting the hypothesis that it is likely to be metabolically expensive to synthesize (*7*). Last, we report that upon starvation for 4 weeks, *Nv1* levels in wild-type animals were down-regulated by ~95% (fig. S5 and table S16; *P* = 0.006), which suggests that Nv1 levels are metered when resources are limited. Therefore, we decided to test whether the production of Nv1 affects the growth and reproduction of *Nematostella.*

**Fig. 4.**
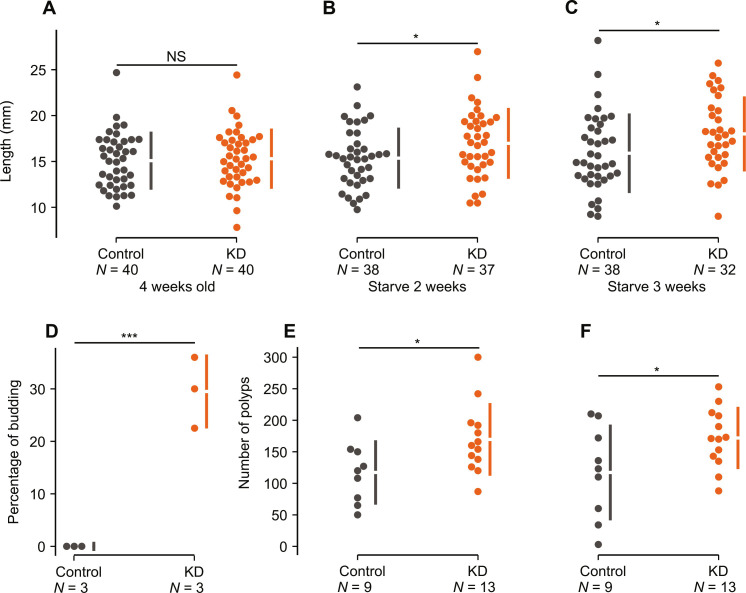
Impact of synthesizing of Nv1 on the physiology and reproduction of *Nematostella*. (**A**) Length (mm) of 4-week-old *Nematostella* fed intensely. (**B**) Length of 6-week-old *Nematostella* following 2 weeks of starvation. (**C**) Length of 7-week-old *Nematostella* following 3 weeks of starvation. (**D**) Percentage of *Nematostella* that went through asexual budding (*n* = 3). (**E**) Number of polyps developed from either control or KD transgenic eggs and fertilized with wild-type sperm. (**F**) Number of successful polyps developed from either control or KD transgenic eggs following 2 weeks of starvation and fertilized with wild-type sperm. Confidence intervals (95%) are indicated by the ends of the vertical error bars. *P < 0.05, ***P < 0.001.

First, we began a feeding trial in which animals from both the control and KD lines were fed an excessive amount of food in the form of crushed brine shrimps. Once the animals reached 4 weeks old, we measured their length and observed no significant difference (Fig. 4A and table S17; *P* = 0.75). Following this, we began starving animals from both lines to determine whether relative growth rates are affected when resources are limited. After 2 weeks of starvation, both lines continued to grow, with KD animals growing significantly more than the control lines (Fig. 4B, and table S17; *P* = 0.046). This significant difference continued after 3 weeks of starvation (Fig. 4C and table S17; *P* = 0.034). However, after 4 weeks of starvation, no differences were observed (table S17; *P* = 0.17). After 4 weeks of starvation, however, we observed an additional nine animals in the containers of the KD line that could have only resulted from asexual reproduction by budding (*17*). A shift in reproductive strategy in the KD lines is supported by our enrichment analysis performed using our RNA-seq results, identifying the up-regulation of genes related to the cell cycle and mitosis in KD *Nematostella* (Fig. 2E and table S7). This suggests that KD animals are undergoing increased rates of mitosis, allowing for faster growth and increased budding.

In *Nematostella*, rapid growth is often associated with asexual reproduction via budding; therefore we tested whether KD animals reproduce more asexually. We find that the KD line asexually buds at significantly higher rates than the control line (Fig. 4D and table S18; *P* = 0.0016). Last, we tested the effect Nv1 synthesis on progeny production through sexual reproduction. We find that females from the KD line produce significantly more polyps than the control line females (Fig. 4E and table S19; *P* = 0.03). We repeated this assay with the addition of starving animals for 2 weeks, as most animals in the field experience prolonged periods without food (table S13C), and find that the KD line still produce significantly more polyps than the control line females (Fig. 4F and table S19; *P* = 0.04). Together with the growth assay results, we report that the synthesis of a single venom component significantly affects the fitness of an animal. This is demonstrated by the fact that when Nv1 levels are reduced, animals can grow faster and reproduce more, both sexually and asexually.

## DISCUSSION

In this study, we unravel how a single toxin can substantially affect a venomous animal’s interspecific interactions, physiology, reproduction, and ultimately fitness. We developed a genetic manipulation technique in *Nematostella* to constitutively knock down multiple copies of *Nv1*, the major adult toxin, and find that these transgenic animals recapitulate the phenotype of a natural population that has undergone a contraction event, resulting in the loss of Nv1. Specifically, the depletion of Nv1 significantly reduces *Nematostella’s* ability to defend itself against grass shrimp (Fig. 3). We also find that *Nematostella* producing wild-type levels of Nv1 can attract a native fish species that is known to prey on grass shrimp, likely acting as an additional form of protection (Figs. 1 and 3). This provides strong evidence of an animal-derived compound initiating a tritrophic interaction. Last, we report that synthesizing massive amounts of Nv1 incurs a significant metabolic burden, as evident from our findings that *Nematostella* producing depleted levels of Nv1 grow faster and reproduce sexually and asexually more (Fig. 4). This work presents a notable example of a significant evolutionary trade-off in *Nematostella*, which must balance the amount of Nv1 synthesized to maintain their ability to sufficiently defend itself against predators with maximizing its growth rate and fecundity.

Trade-offs between defense and growth have been reported extensively in plants and are commonly known as growth-defense trade-offs in which plants adjust growth and defense on the basis of external conditions (*33*). While some of these defensive traits are constitutive, such as the development of a thick physical barrier (for example, bark), other defenses are highly inducible and respond only to the presence of pests resulting in reduced growth. In animals, trade-offs between weaponry and reproduction have also been shown, such as in the horned beetle which balances the investment of horn-size used to obtain mates and testes growth (*34*). Unraveling the genetic basis underlying these trade-offs has been challenging as the traits are often complex and involve large gene networks or vast biosynthetic pathways. Furthermore, many examples of these trade-offs are the result of ecological factors, such as pest presence or nutrient abundance, which further challenge the ability to unravel the evolutionary history of these trade-offs.

Here, we isolated a single heritable locus that underlies the balance between growth, reproduction, and defense. Because of this resolution, we can reconstruct and hypothesize the evolutionary history of *Nv1* in *Nematostella*. Specifically, our findings when combined with previous work showing that *Nv1* copy number varies significantly both within and among *Nematostella* populations (*4*) led us to hypothesize that balancing selection is a major force in shaping the evolution of this locus. In our previous work, we found that the *Nv1* haplotype maintains heterogeneous haplotypes and significant diversity in the copy number of *Nv1* across populations ranging from 8 to 24 copies across Nova Scotia, Maine, New Hampshire, Massachusetts and NC. In addition, variation within each population was also found, with no population reaching fixation. For example, in FL, some individuals have lost *Nv1* completely, whereas others maintain two diploid copies. The role of balancing selection in venom evolution has been proposed in recent studies focusing on snakes, in which the maintenance of genetic diversity better explains the evolution of multiple venom gene families than directional positive selection (*35*, *36*). We hypothesize that balancing selection may indeed be the major force driving the observed variation in *Nv1* copy number. Heterogeneity and diversity of *Nv1* copy number variation within a population may be selected for, as it can help diversify the risk of producing too much or too little Nv1 related to growth/reproduction and defense, respectively. However, if selection pressures act intensely enough to favor faster growth and reproduction at the expense of defense for multiple generations, then this could explain the marked loss of *Nv1* copies in the FL population.

The current results obtained for the differences among *Nematostella* native populations correspond to results from venomous snakes which have also undergone significant expansion and contraction of toxin gene families. A prominent example was shown in the venom of *Trimeresurus flavoviridis* found in Okinawa, which has a divergent venom composition when compared with other surrounding islands (*37*). Snakes from Okinawa were found to have lost the highly active myotoxic phospholipase A2 (PLA2), which correlated with differences in prey items. For example, snakes from Okinawa were found to feed on frogs compared to snakes on the surrounding islands that have myotoxic PLA2 and feed on rats (*37*). Further studies on other viperid snakes have also shown similar correlations between diet and venom in different populations of the same species or closely related species (*29*–*31*). Furthermore, it has also been suggested in snakes that predation and not defense drives the evolution of the venom phenotype; however, notable examples contradict this (*38*). For example, spitting venom from three different lineages of cobras has evolved convergently for defensive purposes and have similar venom profiles (*39*). This strongly supports the notion that defense against predation is also an important selective force driving venom evolution in some cases. Nevertheless, functional proof is lacking in the examples because of the limitations in the genetic toolkit available for these organisms, which is essential as it cannot be excluded that other local adaptations could underlie these differences.

In this study, we find further evidence for the ecological significance of defensive venom using functional and organismal approaches. In *Nematostella*, venom components are present throughout its life history, yet it only becomes predatory after metamorphosis and settlement to become a polyp (*15*, *40*). Together with our findings that the loss of Nv1 is correlated with the significantly reduced ability of *Nematostella* to defend itself against grass shrimp but did not affect the ability to capture prey, this strongly suggests that Nv1 is a defensive venom that has an essential ecological role. This is in agreement with the spatial distribution of *Nv1*, which has higher expression in the physa compared to tentacles, pharynx, and mesenteries (*15*). The physa is located at the aboral pole of *Nematostella* and is typically buried in sediment. When stressed, *Nematostella* will typically retract the tentacles leaving only the body wall exposed. In contrast, tentacles, pharynx, and mesenteries all likely play important roles in catching and digesting prey where *Nv1* levels are found in lower abundances. In contrast, these body regions are abundant with nematocytes (stinging cells) that inject Nv1-less venom using a harpoon-like structure (*41*, *42*). Our results are in accordance with a recent finding that nematocytes in *Nematostella* are functionally specialized for predation, preferentially stinging when exposed to prey signals (*43*). This finding suggests that *Nematostella* separates its venom at both anatomical and cellular levels on the axis of predation and defense.

The separation of venom components and their specialization in predation or defense has been the focus of a significant body of recent research. To date, this research has shown that different venomous animals can produce distinct venom profiles following specific stimuli related to predation or defense. This was demonstrated convincingly in scorpions, cone snails, and an assassin bug (*P. plagipennis*), where they each secreted distinct venom profiles with different activities upon specific stimuli, highlighting that the molecular process for the separation of venom profiles has repeatedly evolved (*8*–*10*, *12*). While these studies showed that different venom profiles with distinct activities are induced by specific stimuli, the functionality was never tested at the organismal level. Here, we experimentally show that toxin expression is separated for functionally distinct roles.

An unexpected finding from our study is that venom may act as a potential chemosensory cue in an aquatic environment. This was previously reported for conotoxin-Tx VIA, a neurotoxin from the venom of a cone snail, that triggers a specific warning response by a prey snail of the genus *Strombus*, causing them to initiate increased escape behavior locomotion (*44*). Together with our results, venom-derived peptides secreted into the aquatic environment may have a more significant impact on ecosystems beyond direct predator-prey interactions such as having additional indirect effects. To date, complex indirect interactions between plants, herbivores, and their natural predators have been reported in detail, often referred to as tritrophic interactions, and have been found to be an integral part of all ecosystems (*45*–*47*). This work has mostly focused on the role of herbivore-induced plant volatiles, as plants can attract predators and parasitoids to defend themselves against herbivores (*45*–*47*). While these plant-derived compounds have been explored from an ecological and mechanistic perspective (*48*–*50*), tritrophic interactions spanning three different animal levels have rarely been studied and are restricted to observational data, often referred to as indirect defense (*26*). Our findings provide evidence that not only a venom component but also a single animal protein can dictate a tritrophic interaction required for defense. Overall, the role of venom in this complex interaction network is a discovery that has not been reported previously. This is an exciting finding as the role of venom in chemical ecology has been largely overlooked and may have more systemic impacts than previously thought, especially in aquatic environments. Together, our work links venom genotype and phenotype to growth, reproduction, and interspecific interactions via an evolutionary trade-off.

## MATERIALS AND METHODS

### * Nv1* copy number variation across *Nematostella* populations

We used quantitative PCR (qPCR) to determine the number of *Nv1* copies in individuals collected from a new location in FL, using methods previously published (*4*). Briefly, DNA for anemones from Nova Scotia, the original FL location, and 10 individuals from a new FL location was isolated with a DNeasy Blood & Tissue kit (Qiagen, USA) using the manufacturer’s protocol. Primers were used for *Nv1* and *Catalase* (*4*). Amplifications were performed in a Bio-Rad CFX96 Touch Real-Time PCR Detection System using the Luna Universal Probe Mix (New England Biolabs, USA). We performed three technical replicates of *Catalase* and *Nv1* for each sample. Our plate setup contained triplicate reactions of a reference sample of known copy number from the original FL population (diploid copy number = 1; derived from genome assembly). The diploid copy number was estimated using the ∆∆Ct approach with Catalase as the single-copy control gene and the original Floridian sample of known copy number as our reference sample.

#### Transgenesis

Two synthetic gBlock gene fragments (Integrated DNA Technologies, Belgium) were designed to target the unique locus encoding Nv1. This included the mOrange2 sequence (*51*) and included sequence taken from the intron that houses miR-2030 (*22*) in *Nematostella* (scaffold_507:85,148-86,273). For our KD line, an artificial miRNA was also included pre-miRNA sequence of Nve-miR-2022 (*22*) and changed the mature miRNA sequence to fully match the *Nv1* transcript, an approach previously described to generate efficient knockdowns (*52*). Following digestion with restriction enzymes, PCR fragments were ligated to a pER242 (*53*) vector containing a TATA box–binding protein (TBP) promoter previously proved to drive ubiquitous expression in *Nematostella* (*24*) and simian virus 40 polyadenylation signal. Plasmids were transformed into the *Escherichia coli* DH5α (New England Biolabs) strain and outsourced for Sanger sequencing (HyLabs, Israel). Each plasmid was subsequently injected into *Nematostella* zygotes along with the yeast meganuclease I–Sce I (New England Biolabs) enable genomic integration (*53*, *54*). Transgenic animals were visualized under an SMZ18 stereomicroscope equipped with a DS-Qi2 camera (Nikon, Japan), and positive animals were reared to the adult stage. At approximately 4 months old, F_0_ individuals were induced for gametes and crossed with wild-type animals to generate F_1_ heterozygotes. Multiple positive F_1_ adults for both lines were then crossed with wild-type animals, and these F_2_ animals were used for subsequent experiments.

#### Knockdown validation

To test the knockdown efficiency of our KD line versus control lines, we performed qPCR and semiquantitative proteomics. Because previous work has shown that *Nv1* expression levels are highest in adult physa (*15*), we chose to test the knockdown efficiency on this tissue type and developmental stage. First, adult animals (>3 months old) were anesthetized with MgCl_2_ and their physa was dissected.

#### RNA extraction

Total RNA was extracted (three replicates made from three individuals each) using TRIzol Reagent (Thermo Fisher Scientific, USA) and following the manufacturer’s protocol. Samples were treated with 2 μl of Turbo DNAse (Thermo Fisher Scientific) and underwent an additional round of extraction using TRIzol Reagent (Thermo Fisher Scientific). A total of 500 ng of RNA was reverse transcribed into cDNA using the iScript cDNA Synthesis Kit (Bio-Rad, USA).

#### Quantitative polymerase chain reaction

Real-time qPCR was performed using Fast SYBR Green Master Mix (Thermo Fisher Scientific) on the StepOnePlus Real-Time PCR System v2.2 (Thermo Fisher Scientific). *Nv1* expression levels were normalized to the NVE5273 gene (ΔCt = Ctreference gene − Ctgene of interest) which has been previously shown to have stable expression level across development and the relative expression calculated 2ΔΔCt method (*15*, *55*). The significance level was calculated by applying paired two-tailed Student’s *t* test to ΔCt values for each of the pairwise comparison between control and KD lines. Plots were made using estimation stats (*56*).

For the quantification of the miRNA in the KD line, we designed the stem-loop primer (*57*, *58*). Next, we used 100 ng of total RNA for cDNA preparation using the SuperScript III Reverse Transcriptase (Thermo Fisher Scientific) and used random primers as a negative control or stem-loop. The presence of the miRNA primers was determined by using end-point PCR (*59*), using 2 μl of cDNA as template, miRNAs-specific forward primer and stem-loop–specific reverse primer and running the PCR at 94°C for 2 min, followed by 35 cycles of 94°C for 15 s and 60°C for 1 min (*58*).

#### Sample preparation for mass spectrometry analysis

Dissected adult physa was also used for semiquantitative tandem mass spectrometry (MS/MS) analysis and performed using adults (five replicates, each made of three individuals) from both control and KD lines. Samples were snap frozen and lysed using 8 M urea and 400 mM ammonium bicarbonate solution. Lysed samples were centrifuged (22,000*g*, 20 min, 4°C), and the supernatant was collected. Protein concentrations were measured with the BCA Protein Assay Kit (Thermo Fisher Scientific).

Protein samples were denatured and reduced in 100 μl of 8 M urea, 10 mM dithiothreitol, and 25 mM tris-HCl (pH 8.0) for 30 min. Iodoacetamide (55 mM) was added, and samples were incubated in the dark for 30 min. Urea was diluted by the addition of 8 volumes of 25 mM tris-HCl (pH 8.0) followed by the addition of sequencing-grade modified trypsin (Promega Corp., USA) (0.4 μg per sample) and incubated overnight at 37°C with gentle agitation. The resulting peptides were acidified by the addition of 30 μl of 10% formic acid (FA) and desalted on C18 home-made stage tips. Peptides were eluted using 80% acetonitrile and 0.1% FA and dried. Peptides were reconstituted in 0.1% FA, and peptide concentration was determined by absorbance at 280 nm.

#### Nano–liquid chromatography–MS/MS analysis

MS analysis was performed using a Q Exactive HF mass spectrometer (Thermo Fisher Scientific) coupled on-line to a nanoflow ultrahigh performance liquid chromatography (UHPLC) instrument, Ultimate 3000 Dionex (Thermo Fisher Scientific). From each sample, 0.35 μg of peptides was injected. Peptides were separated over a 107-min acetonitrile gradient (4 to 50%) at a flow rate of 0.15 μl/min on a reverse phase 25-cm-long C18 column [75-μm inside diameter (ID), 2 μm, 100 Å, Thermo PepMapRSLC]. Survey scans [300 to 1650 mass/charge ratio (*m/z*); target value, 3 × 10^6^ charges; maximum ion injection time, 20 ms] were acquired and followed by higher energy collisional dissociation (HCD)–based fragmentation (normalized collision energy, 27). A resolution of 60,000 was used for survey scans and up to 15 dynamically chosen most abundant precursor ions, with “peptide preferable” profile were fragmented (isolation window, 1.6 *m/z*). The MS/MS scans were acquired at a resolution of 15,000 (target value, 1 × 10^5^ charges; maximum ion injection times, 25 ms). Dynamic exclusion was 20 s. Data were acquired using Xcalibur software (Thermo Fisher Scientific).

#### MS data analysis

Mass spectra data were processed using the MaxQuant computational platform, version 2.0.3.0. Peak lists were searched against an NVE FASTA sequence database with the addition of the mOrange2 protein sequence (https://figshare.com/articles/dataset/Nematostella_vectensis_transcriptome_and_gene_models_v2_0/807696). The search included cysteine carbamidomethylation as a fixed modification, N-terminal acetylation, and oxidation of methionine as variable modifications and allowed up to two miscleavages. The “match-between-runs” option was used for lysate samples. For the water sample, the “dependent peptides” option was used. Peptides with a length of at least seven amino acids were considered and the required false discovery rate (FDR) was set to 1% at the peptide and protein level. Relative protein quantification in MaxQuant was performed using the label-free quantification (LFQ) algorithm for lysate samples only (*60*). MaxLFQ allows accurate proteome-wide LFQ by delayed normalization and maximal peptide ratio extraction (*60*). Statistical analysis (*n* = 5) was performed using the Perseus statistical package, Version 1.6.2.2121. Only those proteins for which at least three valid LFQ values were obtained in at least one sample group were accepted and log_2_ transformed. Statistical analysis by Student’s *t* test and permutation-based FDR (*P* < 0.05). After the application of this filter, a random value was substituted for proteins for which LFQ could not be determined (“Imputation” function of Perseus). The imputed values were in the range of 10% of the median value of all the proteins in the sample and allowed the calculation of *P* values.

#### RNA sequencing

To further test the knockdown efficiency as well as if any other toxins are compensating for the reduction in *Nv1* across the whole organism, we performed RNA sequencing and differential gene expression. RNA was extracted as described above with the exception of using juvenile whole organisms (five individuals at 2 months old). Total RNA quality was assessed using Bioanalyzer Nanochip (Agilent, USA) with all samples having RNA Integrity Number (RIN) > 7. Libraries were generated from 100 ng of RNA using NEBNext Ultra II Directional RNA Library Prep Kit (New England Biolabs) for Illumina and sequenced using a 75-bp single-end sequencing on a NextSeq 500 machine (Illumina, USA).

Raw reads were trimmed and quality filtered using Trimmomatic (*61*). Reads were mapped to a modified *Nematostella* transcriptome (https://figshare.com/articles/dataset/Nematostella_vectensis_transcriptome_and_gene_models_v2_0/807696) with *Nv1* transcripts collapsed into a single contig. Mapping was performed using Bowtie2 (*62*), and the gene counts were quantified using RNA-Seq by Expectation Maximization (*63*). Differential expression analyses were performed using scripts from Trinity (*64*) using both DESeq v2.139 (*65*) and edgeR v3.167 (*66*). Differentially expressed genes were defined by FDR < 0.01 and log_2_ fold change ≥0.8. Genes identified by both methods were considered as differentially expressed. Biological replicates were quality checked for batch effect using sample correlation and principal component analysis. Volcano plots were generated using EnhancedVolcano (*67*). Transcriptome was annotated using BLASTx against the swiss-prot database (accessed on 4 January 2023) and enriched Gene Ontology (GO) groups identified using GOseq (*68*) Bioconductor package implemented in the in-built Trinity pipeline (*64*). An FDR cutoff of 0.05 was considered significant for the enriched or depleted GO terms.

#### Interspecific interactions

##### Animal care

*Nematostella* embryos, larvae, and juveniles were grown in the dark at 22°C in 15‰ ASW, whereas polyps were grown at 18°C. All polyps were fed with *Artemia salina* nauplii three times a week. The induction of gamete spawning was performed as previously described (*69*). The gelatinous egg sack was removed using 3% l-cysteine (Merck Millipore) and followed by microinjection of the plasmids. All *Nematostella* individuals used in this study belonged to the common laboratory strain originating from Rhode River MD (*17*).

Fertilized mummichogs (*F. heteroclitus)* eggs from Kings Creek, VA (37°18′16.2″N 76°24 58.9″W) and Scorton Creek, MA (41°43′52.1″N 70°24′51.3″W) were provided by R. Trevisan (Duke University) and D. Nacci (Environmental Protection Agency), respectively. They were kept in 15‰ ASW at room temperature until hatching (around 2 to 3 weeks) and used immediately for behavioral analyses. Experiments on mummichogs were performed under permit no. 17–018 granted by the Institutional Animal Care and Use Committee (IACUC) at the University of North Carolina at Charlotte (IACUC-22-041 (UNC Charlotte) according to ethical regulations of Office of Laboratory Animal Welfare (National Institutes of Health, USA).

The first batch of grass shrimps (*P. pugio*) was collected at an estuary near Georgetown, SC (33°21′01.0″N 79°11′26.1″W). Animals were transported to the laboratory and kept in 15‰ ASW in recirculating aquaria until use. Interaction experiments were conducted in 15‰ ASW containers. Grass shrimps were fed every day with TetraMin tropical fish food (Tetra Holding, USA). Mummichogs were fed twice daily with *Artemia* reared in the laboratory.

##### Nematostella interactions with predators

To test the interaction of grass shrimp with *Nematostella*, all animals were starved for 48 hours before the experiment. Grass shrimp and *Nematostella* from different lines were placed in a container (27.5 cm in length, 8 cm in width, and 9 cm in height) with 1000 to 1200 ml of 15‰ ASW and onyx sand premium natural substrate (Seachem, USA) and separated by a divider for 30 min before recording the experiment for 15 min using Moticam 580 (Motic, China). Grass shrimp were gently introduced, and the recording started. Videos were exported and a 15-grid key was overlayed on top of the recording using Adobe Premiere Pro. Scoring was performed based on grid locations of grass shrimp every 10 s (fig. S6). At each time interval, if the predator was touching the sea anemone a score of 0.6 was given, if the predator was within a single grid away a score of 0.3 was given, and all other location were given a score of 0.1. For grass shrimp experiments, we examined multiple transgenic lines. This included laboratory strain wild-type, TBP::mCherry, control, and KD lines. One-way ANOVA and Tukey post hoc test analyses were performed to determine significance in a pairwise manner. No differences were observed between WT, mCherry, and control lines. Experiments comparing control and KD lines did not include WT and mCherry lines. Grass shrimp interactions were also performed for the comparison of NC and FL animals and were analyzed using a two-tailed Student’s *t* test. Interspecific interactions between *Nematostella*- mummichog were also performed as above with the exception of a 15-min acclimation, a 10-min recording time, and the use of a different substrate (White Coral Sand, Nature’s Ocean Premium Marine Substrates, USA) to allow better visualization of the mummichogs.

##### Exposure of young mummichogs to *Nematostella* treated water behavioral assay

To test whether Nv1 is secreted into the water and affects fish behavior, we performed an additional behavioral analysis of mummichogs’ movement. First, to detect whether Nv1 is being secreted into the water, we performed an MS/MS analysis on water incubated with *Nematostella* overnight. Twelve wild-type females were placed in 42 ml of 15‰ ASW overnight (16 hours). Water was then collected and filtered using a 0.22-μm filter (Merck Millipore, USA) to remove any debris and released nematocytes. Filtered treated water was then concentrated first using Amicon Ultra-15 Centrifugal Filter Unit 3 kda (Merck Millipore) at 12°C and 2686*g* to a final volume of 0.5 ml. We further concentrated this down to 40 μl using the Amicon Ultra-0.5 Centrifugal Filter Unit (Merck Millipore) at 12°C and 4000*g* and submitted it for MS analysis.

MS analysis was performed using a Q Exactive-Plus mass spectrometer (Thermo Fisher Scientific) coupled on-line to a nanoflow UHPLC instrument, Ultimate 3000 Dionex (Thermo Fisher Scientific). Approximately 0.45 μg of peptides was injected. Peptides were separated over a 52-min acetonitrile gradient (4 to 50%) at a flow rate of 0.15 μl/min on a reverse phase 25-cm-long C18 column (75-μm ID, 2 μm, 100 Å, Thermo PepMapRSLC). Survey scans (380 to 2000 *m/z*; target value, 3 × 10^6^ charges; maximum ion injection time, 50 ms) were acquired and followed by HCD-based fragmentation (normalized collision energy, 25). A resolution of 70,000 was used for survey scans and up to 15 dynamically chosen most abundant precursor ions, with “peptide preferable” profile were fragmented (isolation window, 1.8 *m/z*). The MS/MS scans were acquired at a resolution of 17,500 (target value 1 × 10^−5^ charges; maximum ion injection times, 121 ms). Dynamic exclusion was 60 s. Data were acquired using Xcalibur software (Thermo Fisher Scientific). MS data analysis was performed as described above.

Next, we performed a behavior analysis of mummichogs in treated water coming from either control or KD animals. Before the behavioral assay of mummichogs, KD and control anemones were starved for 48 hours. The behavioral assays took place in six-well plates where each well contained 7 ml of 15‰ ASW. Two anemones were placed in each well according to the trial taking place (KD or control) for 24 hours. Anemones were removed and the water was syringe filtered with a 0.22-μm Millex GP Filter unit (Merck Millipore). After filtration, we began an acclimation step in a new six-well plate filled with 15‰ ASW. We created a transfer basket by modifying 40-μm easy strainers by cutting off the handles and placing a single strainer in each well. Young mummichogs were collected, and a single fish larva was placed in a transfer basket in the acclimation well plate. Fish larvae were acclimated in the room for 10 min and were then transferred to the experimental well plate that contained filtered anemone water in a DanioVision recording chamber (Noldus Information Technology, Netherlands). Video recordings were captured for 15 min, where recordings began before the transfer. We performed 9 replicates of fish exposed to KD anemone water and control anemone water, along with 10 replicates of a control that contained only 15‰ ASW (no exposure to anemones). We then used EthoVision XT 9 and XT 12 software (Noldus Information Technology) to track fish movement which was used in the analysis. Movement was then recorded using DanioVison (Noldus Information Technology) every 0.1 s, where movement <0.1 mm was considered noise and removed. Distances were then used to perform a one-tailed Student’s *t* test as previous work has shown that zebrafish move more when incubated with the recombinant Nv1 compared to bovine serum albumin (*13*). FDR corrections were performed to correct for multiple comparisons.

##### Exposure of young zebrafish to Nematostella treated water behavioral assay

To detect whether secreted Nv1 affects other fish behavior, we performed an additional movement assay using zebrafish. First, 28 females were placed in 100 ml of 15‰ ASW overnight (16 hours). Water was then collected and filtered using a 0.22-μm filter (Merck Millipore) to remove any debris and released nematocytes. Filtered treated water was then concentrated 4× using Amicon Ultra-15 Centrifugal Filter Unit 3 kda (Merck Millipore) at 12°C and 2686*g* to a final volume of 0.5 ml. We then performed buffer exchange and used E3 medium and repeated this process three additional times.

Wild-type adult zebrafish were intercrossed and their progeny were kept under light-dark (LD) cycle. At 14 days after fertilization, the zebrafish were placed in a 48-well plate, alternating between control and KD water. The plates were placed in the DanioVision tracking system (Noldus Information Technology) and allowed to acclimate for 15 min before recording their activity. The light intensity in the tracking system was set at 70 LUX (25% in the operating software) for all experiments. To observe their responses to LD transitions, the zebrafish experienced 3 cycles of 30 min of light followed by 30 min of darkness. Each experiment involved four independent assays, which were recorded and analyzed using the EthoVision XT 9 and XT 12 software (Noldus Information Technology), as previously described (*70*). The data analysis for total activity was conducted according to previously described threshold parameters (*70*). Zebrafish protocol was reviewed and approved by the Bar-Ilan University Bioethics Committee.

##### Nematostella interactions with prey

To analyze the gut contents of *Nematostella* we used whole-body genomic DNA extractions using a protocol described in our previous study (*4*). Total genomic DNA was extracted using the AllPrep DNA/RNA Kit (Qiagen, USA) for 10 or more individuals across five locations spanning the Atlantic coast of North America (Cresent Beach, Nova Scotia; Saco, Maine; Wallis Sand, New Hampshire; Sippewissett, Massachusetts; Ft. Fisher, NC) during the months of March, June, and September in 2016. To target gut contents we used the primers (*71*) (LCO1490:GGTCAACAAATCATAAAGATATTGG and HC02198:TAAACTTCAGGGTGACCAAAAAATCA) to amplify the Cytochrome C Oxidase subunit 1 (CO1) along with the adapter overhang for Nextera Indexing. Total genomic DNA extractions and PCR amplifications were conducted similarly to previously reported (*4*). Briefly, PCRs with modified adapters were performed using HiFi HotStart Ready Mix (Kappa Biosciences, Germany) with the following conditions: 95°C for 3 min; 8 (95°C for 30 s, 40°C for 30 s, 72°C for 1 min), 72°C for 5 min. Successful amplification was checked using the same approach, with an increase in cycles (35×) to visually inspect via gel electrophoresis. Samples without visible bands were still included for sequencing to potentially sequence low-abundance PCR products. Libraries were prepared for sequencing using the MiSeq Reagent Kit v3 (600 cycles) (MS-102-3003) and indexed using the Nextera XT Index Kit V2 and sequenced alongside 5% PhiX. Overlapping reads were joined using BBMerge (*72*) (Version 38.84), and duplicate sequences were counted within Geneious Prime 2023.1.1.

A CO1 database was constructed using sequences retrieved from National Center for Biotechnology Information (NCBI) Genbank using the Entrez Direct (Edirect) utility (accessed on 10 May 2023), specifically nucleotide sequences that contained the term cox1. MiSeq sequences were then searched against the custom CO1 database using blastn, with the top blast hit retained as a candidate sequence for each sampling locality. A series of custom python scripts (https://github.com/JasonMacrander/Stella_Venom) were used to identify sequence abundance and taxonomic diversity across sites.

Prey immobilization and consumption were compared between control and KD anemones with observational feeding experiments. We used two copepod species (*T. californicus* and *T. biminiensis*) purchased from AlgaeBarn (USA) and differ in size but are representative of the small arthropods that compose a significant portion of the anemone’s natural diet (*73*). *N. vectensis* juveniles for these experiments resulted from crosses of multiple wild-type females with a transgenic male from either control or KD lines. Positive polyps were then picked 10 days after fertilization. Newly settled 4-tentacle juveniles were fed freshly hatched *Artemia* nauplii for 2 to 3 weeks to increase their size to 8- to 12-tentacle juveniles. This size was determined in preliminary experiments to be necessary for the efficient capture of these two copepod species. Smaller juveniles were capable of capturing copepods but encounter rates were too low for reliable comparisons of capture and consumption. In preparation for the observations, anemones were fed freshly hatched *Artemia* 48 hours before the experiment and then placed in new 15‰ ASW. Fifteen anemones from each line (KD and control) were placed individually in a single well of a flat bottom 96-well plate with 200 μl of seawater. A single copepod was introduced into the well and observed using a stereomicroscope (Leica M80, Germany). The time to immobilization was scored as the time until the copepod ceased movement. The time to consumption was measured as the time until the copepod was fully ingested into the mouth. Times were recorded to the nearest second. The recorder was blind to the treatment group at the time of experimentation and observation. Statistical comparisons for time to immobilization and time to consumption were completed using Student’s *t* test and a *P* value of 0.05 was used to assess statistical significance.

We further tested the role of Nv1 in predation of a vertebrate. We placed mummichog larvae with *Nematostella* adults in a small environment to measure capture efficiency for the KD, control, FL, and NC individuals. To do this, we used a 15-ml Falcon tube, which contained 2 ml of sediment and 6 ml of 15‰ ASW. Sea anemones and fish were allowed to first acclimate for 15 min. Fish were then introduced into the tube with the sea anemone in sediment, and capture and consumption were recorded over 15 min. For each line, we had 10 replicates.

### Physiological and reproduction assays

We next aimed to test the impact of synthesizing Nv1 on the physiology of *Nematostella*. To do this, we first investigated the growth rate of animals with our control lines with wild-type Nv1 levels against our KD line with depleted Nv1 levels. Specifically, 40 positive animals per line were picked and grown in small plates with 20 animals per plate and grown at 25°C. Each plate of polyps was fed with 100 μl of highly concentrated artemia that was ground using a micro pestle in a randomized manner four times per week and water changed after a minimum of 3 hours until animals were 4 weeks old. Animals were visualized under the SMZ18 stereomicroscope equipped with a DS-Qi2 camera (Nikon) and measured using Fiji (*74*). Animals were then starved with water changes once a week and measured as above. All measurements were performed blinded and used to perform Student’s *t* test.

Next, we tested the difference in asexual reproduction rates between control and KD lines. To do this, we tracked the number of polyps for 1 month at three different ages, ranging from 1 month old (*n* = 40), 2 months old (*n* = 25), and 3 months old (*n* = 25). To test differences in sexual reproduction, we sorted a cohort of 36 animals from both control and KD lines. Females were identified for both control (9) and KD (13) lines following induction and gamete identification. Animals were induced again 2 weeks later to get them in cycle, and on the third induction, the egg packages from control and KD lines were fertilized with wild-type sperm. Once the animals reached 2 weeks old, polyps were anesthetized using MgCl_2_ and counted. Animal care and polyp quantification was performed blinded.

To understand the impact of starvation on the expression of *Nv1*, we performed a qPCR analysis. Adult wild-type females were first acclimated to being fed three times a week for 1 month and grown in six-well plates. After acclimation, nine females were starved, while nine were fed under the same conditions and after 4 weeks, physa was removed from all animals and snap frozen (three replicates with three individuals per replicate). RNA extraction, cDNA synthesis, and qPCR analysis were performed as described above.
