# Supplementary material for: Venom trade-off shapes interspecific interactions, physiology, and reproduction
Source: Sci Adv. 2024 Mar 13;10(11):eadk3870. doi: 10.1126/sciadv.adk3870 (PMC10936874; doi:10.1126/sciadv.adk3870)
Supplement: Supplementary file 1 — Figs. S1 to S6 Legends for tables S1 to S19 Legends for movies S1 to S3 [file sciadv.adk3870_sm.pdf]

Science Advances
AAAS

Supplementary Materials for

Venom trade-off shapes interspecific interactions, physiology, and reproduction

Joachim M. Surm *et al.*

Corresponding author: Joachim M. Surm, joachim.surm@mail.huji.ac.il;
Yehu Moran, yehu.moran@mail.huji.ac.il

Sci. Adv. **10**, eadk3870 (2024)
DOI: 10.1126/sciadv.adk3870

The PDF file includes:

Figs. S1 to S6
Legends for tables S1 to S19
Legends for movies S1 to S3

Other Supplementary Material for this manuscript includes the following:

Tables S1 to S19
Movies S1 to S3

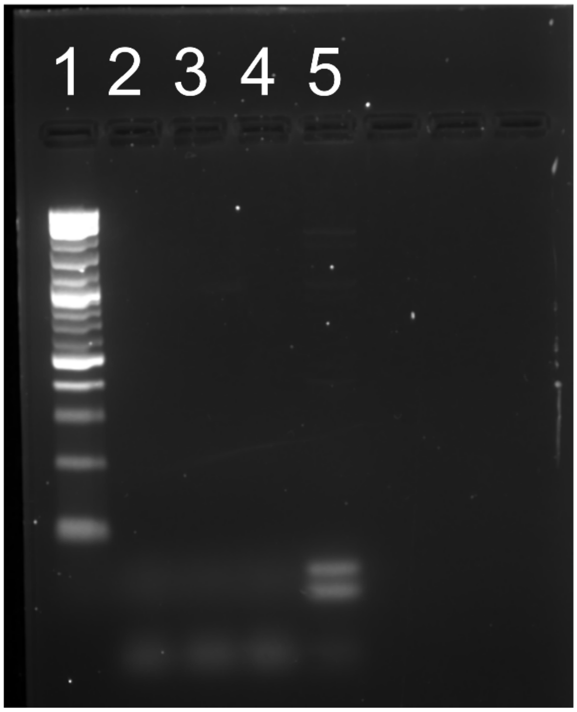

Fig. S1. Validation of miRNA in transgenic KD animals. 2.5% agarose gel picture of miRNA. Lane 1 ladder, lane 2 control cDNA using random primers, lane 3 control cDNA using Nv1-mimiR stem-loop, lane 4 KD cDNA using random primers, lane 5 control cDNA using Nv1-mimiR stem-loop.

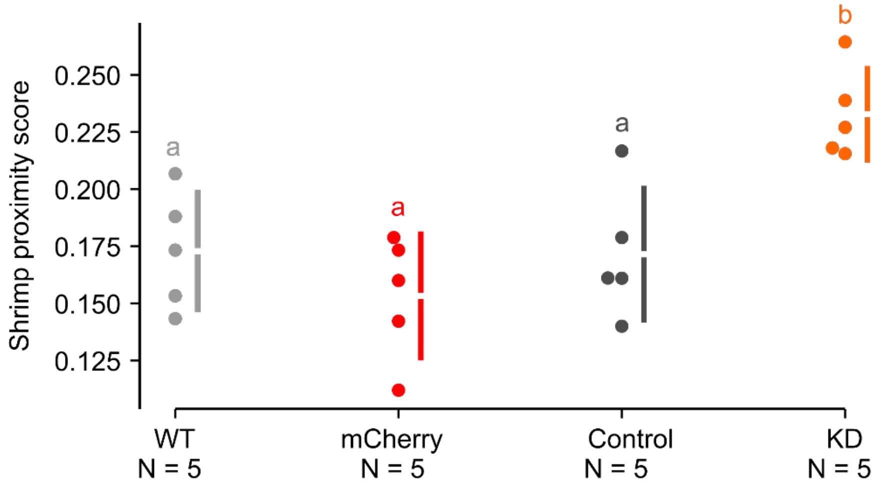

Fig. S2. Shrimp interaction against multiple different *Nematostella* lines. Average weighted score of grass shrimp proximity to *Nematostella* from different lines, including wild-type (WT), TBP::mCherry, control and KD lines. The letters above dot plots indicate the results of a Tukey–Kramer post hoc test, with lines showing significant differences (P-value < 0.05 for pairwise comparisons) unless they share the same letter. 95% confidence intervals are indicated by the ends of the vertical error bars.

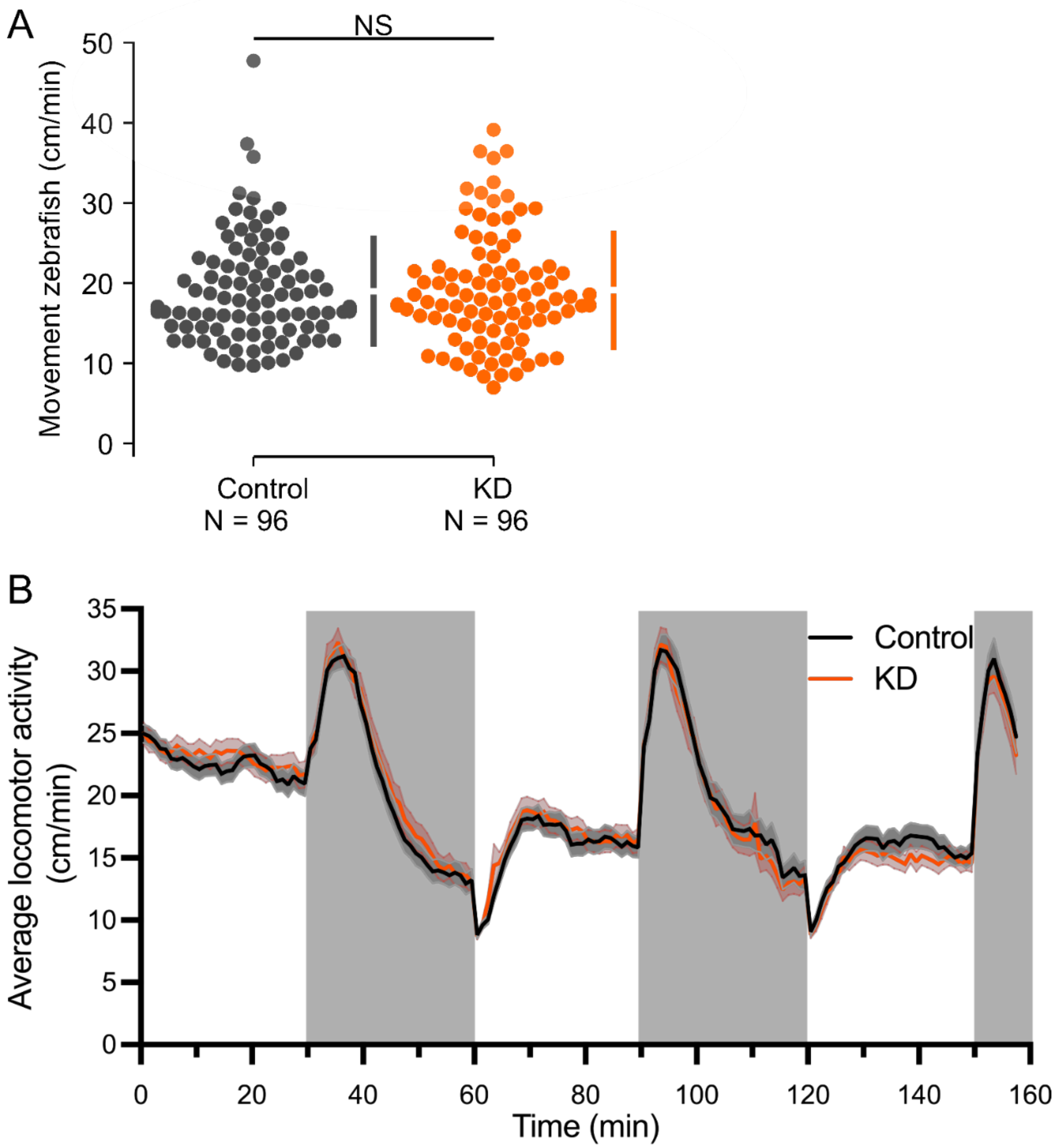

Fig. S3. Tracking the movement of zebrafish. A) Average zebrafish movement (cm/min) over 180minutes in treated water coming from either KD or control *Nematostella* lines. A)95% confidence intervals are indicated by the ends of the vertical error bars. B) Average zebrafish movement for each minute, light and dark conditions with in dark conditions highlighted in grey.

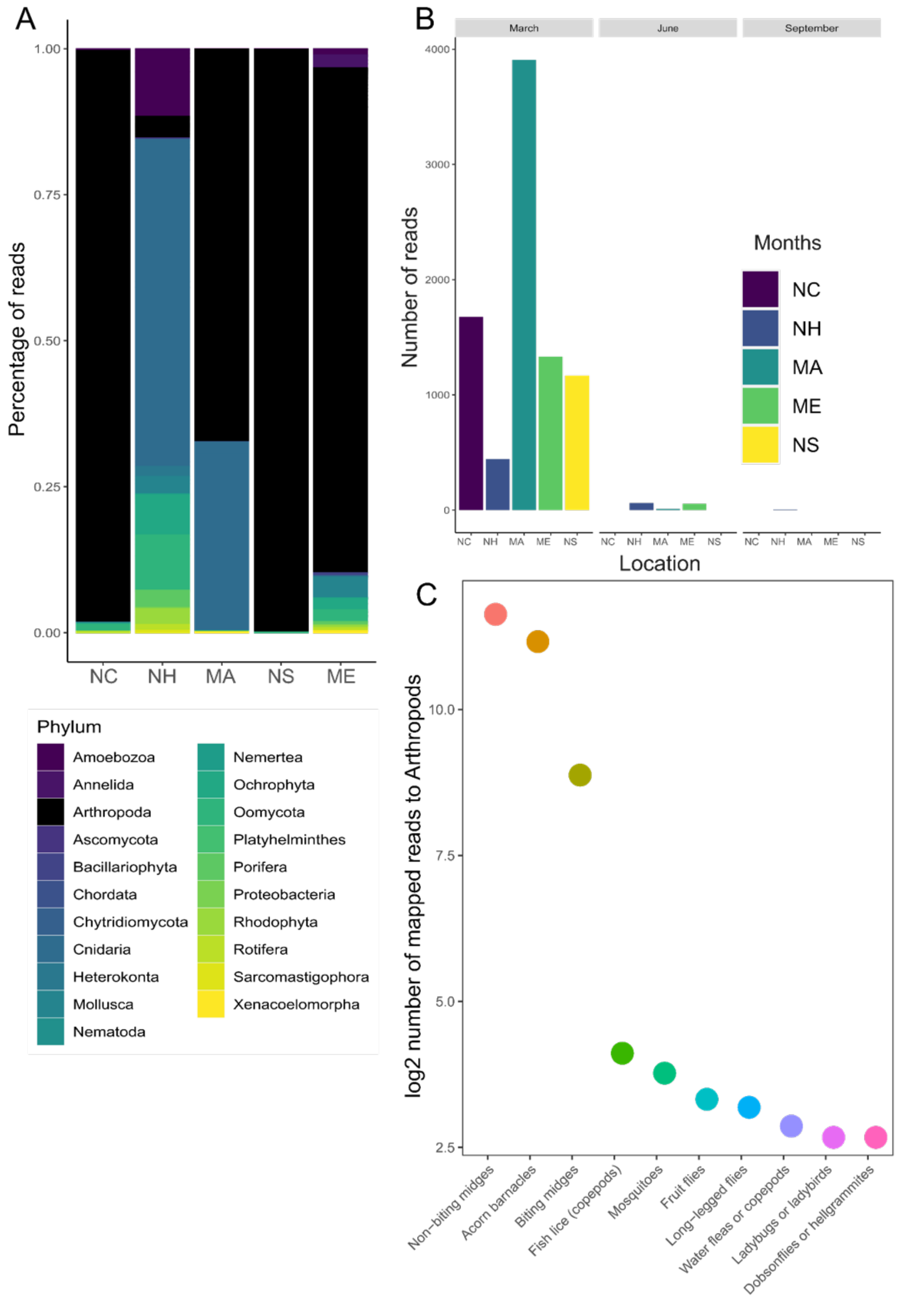

Fig. S4. Metagenomics of gut content of *Nematostella* across different populations in March, June and September 2016. A) Percentage of reads from each population mapping at the phylum level, sequences mapping to Arthropod highlighted in black. B) Number of mapped reads in different populations across March, June and September. C) Cumulative number of reads log2 normalized from each population mapping to different arthropod species. North Carolina (NC), New Hampshire (NH), Massachusetts (MA), Nova Scotia (NS) and Maine (ME).

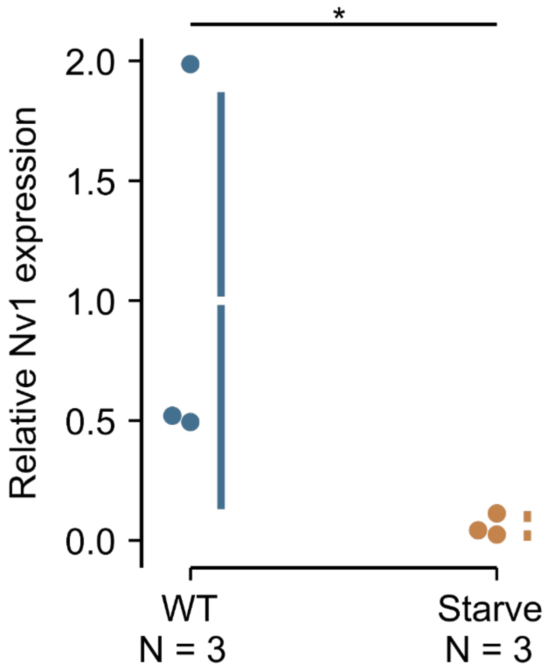

Fig. S5. Gene expression dynamics of Nv1 following starvation. RT-qPCR measuring the expression of Nv1. Plotted values are mean ΔCT comparing wild-type to starved *Nematostella*.. 95% confidence interval is indicated by the ends of the vertical error bar.

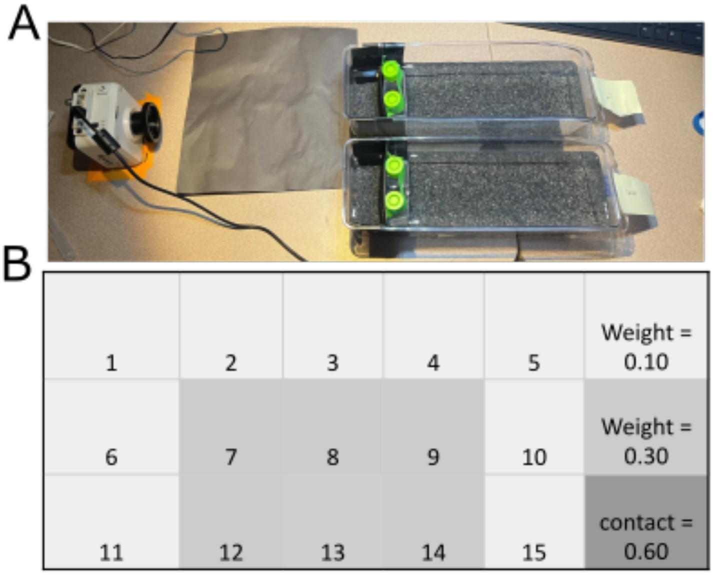

Fig. S6. Setup for interspecific interaction between Nematostella and predators. (A) representation of the setup the container used. (B) scoring scheme used to quantify the proximity of predators to different lines of *Nematostella*. Scoring was performed based on grid locations of grass shrimp every 10 seconds. At each time interval, if the predator was touching the sea anemone a score of 0.6 was given, if the predator was within a single grid away a score of 0.3 was given, and all other location were given a score of 0.1.

Table S1.

Meta-analysis nCounter data of Nv1 expression and housekeeping gene HKG4 across populations from different states of USA. Maryland (MD), North Carolina (NC), Florida (FL). A) Normalized nCounter data of Nv1 expression. C) ANOVA statistics included. B) Nv1 diploid copy number from Nova Scotia and two distinct locations from Florida.

Table S2.

Average weighted score of grass shrimp proximity to *Nematostella* from North Carolina (NC) and Florida (FL). Student's t-test (two-tail) also included.

Table S3.

Average weighted score of mummichog proximity to *Nematostella* against North Carolina (NC) and Florida (FL). Student's t-test (two-tail) also included.

Table S4.

A) Relative quantification (ΔCT) of Nv1 expression from control and KD lines. Student's t-test (two-tail) also included. B) Normalized relative quantification (ΔΔCT) of Nv1 expression from control and KD lines. C) Fold change difference Nv1 expression between control and KD lines. D) Knockdown percentage of Nv1 expression in KD line.

Table S5.

Perseus statistical analysis performed using semi-quantitative proteomic results from control and KD lines of *Nematostella vectensis*.

Table S6.

RNA-seq from control and KD lines of *Nematostella vectensis* with a log2foldchange of 0.8 and *P*-value 0.01. A) DeSeq2 result from genes upregulated in KD line. B) edgeR result from genes upregulated in KD line. C) DeSeq2 result from genes upregulated in control line. D) edgeR result from genes upregulated in control line

Table S7.

Go-term enrichment analysis using clusterProfile (A) and GoSeq (B)/

Table S8.

Average weighted score of grass shrimp proximity to *Nematostella* from wild-type (WT), TBP::mCherry (mCherry), control and KD lines. ANOVA statistics and Tukey HSD / Tukey Kramer included.

Table S9.

Average weighted score of mummichog proximity to *Nematostella* from control and KD line. Student's t-test (two-tail) also included.

Table S10.

Semi-quantitative proteomic result of treated water coming from wild-type *Nematostella*.

Table S11.

Average movement of mummichogs in water coming from untreated water (water), control and KD treated water. Student's t-test (one-tail) and FDR correction also included.

Table S12.

Average movement of zebrafish in water coming from control and KD treated water. Student's t-test (one-tail) also included.

Table S13

Metagenomics analysis of gut contents of *Nematostella* from different population. North Carolina (NC), New Hampshire (NH), Massachusetts (MA), Nova Scotia (NS) and Maine (ME). A) Number of CO1 sequences mapping back to animals in March 2016 at the phylum, order and family level. B) Total number of sequences from populations mapping in different months including March, June and September. C) Number of individuals from different populations that had no reads mapping to database.

Table S14.

A) Time (sec) taken for *Nematostella* to capture and ingest *Tisbe biminiensis* in control and KD lines. B) Time (sec) taken for *Nematostella* to capture and ingest *Tigriopus californicus* in control and KD lines.

Table S15.

Time (sec) taken for *Nematostella* to capture and ingest mummichogs in NC, FL, control and KD lines.

Table S16.

A) Relative quantification (ΔCT) of Nv1 expression in adult wild-type females (WT) and starved adult wild-type females. Student's t-test (two-tail) also included. B) Normalized relative quantification (ΔΔCT) of Nv1 expression from control and KD lines. C) Fold change difference Nv1 expression between control and KD lines. D) Knockdown percentage of Nv1 expression in KD line.

Table S17.

Growth assay of *Nematostella* following intensive feeding and measured (mm) at 4 weeks old followed by 2, 3 and 4 weeks of starvation for control and KD lines. Student's t-test (two-tail) also included.

Table S18.

Asexual reproduction rates from three independent observations measured as percentage of new polyps by original number of polyps for control and KD lines. Student's t-test (two-tail) also included.

Table S19.

A) Sexual reproduction rates measured as number of successful polyp development after 14dpf in control and KD lines. B) Sexual reproduction following 2 weeks of starvation in control and KD lines.

Movie S1.
Movie showing shrimp exhibiting severe recoil following a touch with a control animal.

Movie S2.
Movie showing shrimp exhibiting avoidance in swimming behavior when placed with control animal.

Movie S3.
Movie showing shrimp actively engaging with KD animal as well as some feeding behaviour.
